# Sensory sweetness and sourness interactive response of sucrose-citric acid mixture based on synergy and antagonism

**DOI:** 10.1038/s41538-022-00148-0

**Published:** 2022-07-19

**Authors:** Yuezhong Mao, Shiyi Tian, Yumei Qin, Shiwen Chen

**Affiliations:** School of Food Science and Biotechnology, Zhejiang GongShang University, Zhejiang, 310018 China

**Keywords:** Human behaviour, Weight management, Dietary carbohydrates

## Abstract

The clarity of taste sensation interaction is a key basis for promoting the food sensory science research and its application to the beverage and food additive industries. This study explored the synergy and antagonism effect of sucrose-citric acid mixture and established an optimized method to determine the human sweetness and sourness interactive response. Sucrose-citric acid mixtures were evaluated by the “close type” question. According to the sensory difference strength curves and Weber–Fechner law, citric acid increased the sucrose’s absolute threshold (0.424–0.624%) and weber fraction (20.5–33.0%). Meanwhile, sucrose increased citric acid’s absolute threshold (0.0057–0.0082%) and decreased its weber fraction (17.96–9.53%). By fitting absolute threshold and weber fraction variation equations, the sweet–sour taste sensory strength variation models (SSTVM) were derived, which could be used to explain the synergy and antagonism effect of sweet–sour taste. According to the SSTVM, the interactive response to sweet–sour taste could be quantitatively calculated. The high coincidence between SSTVM and human evaluation (1.02% of relative error) indicated that it could be applied in the food industry, health management, and intelligent sensory science.

## Introduction

Taste sensation is one of the most important sensory qualities of food, including sour, sweet, bitter, salty, umami and some other tastes^1^. The sensory characteristic of human’s response to food, a kind of complex component system, is usually a comprehensive response of multiple taste sensations. This comprehensive taste response is mainly caused by the interaction results of multiple taste sensations through the synergism and antagonism effect^2–4^. The synergism and antagonism effect between different taste sensations has become an essential core basis for humans to experience and enjoy the variety of taste flavors and qualities of food^5^.

In recent years, many studies are focused on the synergism and antagonism effect between two taste sensations. Woskow lab discovered that the umami taste can enhance the sweet and salty taste strength under the medium concentration, while the sour and bitter taste strength were suppressed^6^. Kemp lab discovered that, under medium and high concentration levels, monosodium glutamate could suppress the strength of sweet and bitter taste. And the salty taste strength of sodium chloride would be enhanced by a high concentration of monosodium glutamate^7^. Breslin, Calvino, Curtis, Prescott and Schiffman labs’ research results showed that a low strength of sweet taste had both enhanced and suppressed effects on different taste sensations. At the medium or high strength level, the sweet taste would usually suppress other taste sensations. In addition, the interaction between sweet and bitter was symmetrically suppressed^8–13^. Stevens and Keast labs discovered a low concentration of salty substance could enhance the sweet taste and be suppressed by sweet taste at the medium concentration. And salty taste could suppress the bitter taste and was not suppressed by the bitter taste^14,15^. Tian lab discovered that the perceived sweet and bitter taste strength suppressed each other, while the perceived sour and salty taste strength enhanced each other^16^. Based on the above mentioned research results, it can be found that the studies on taste sensory interaction are almost focused on the trend of synergism and antagonism effect when two taste sensations coexist. However, the detailed quantitative equation or rule of the strength variation has not been deeply explored.

In fact, in experimental psychophysical studies of food sensory area, the taste sensation strength rule can be described and explained by the equation between taste stimulus concentration and psychological sensation caused by taste stimulus, including the Weber–Fechner law and Stevens law^17^. Schutz, Moskowitz and Mao labs used the sweet and sour taste sensation as a research subject, based on the Weber–Fechner law and Stevens law, explored the relationship between the sweet–sour taste stimulus and the weber fraction and stevens index^18–20^. Based on Stevens law, Liu lab determined the umami sensation in puffer fish, grass carp, and their soup by using the method of quantity estimation and established the corresponding mathematical equation^21^. The above studies have established stimulus concentration and psychological sensation strength equation which can well explain the strength variation rules for a single taste sensation. However, the variation rule of psychophysical sensation strength of synergism and antagonism effect for two different taste sensations also has not been systematically studied. It can be believed that the clarity of the variation rule will be a key basis for promoting the food sensory science research and its application to the beverage and food additive industries.

In the previous work, we used “close type” question^18,22^, based on the triangle test method and paired comparison test method, to establish an optimized human sensory sweetness and sourness method. In this study, sucrose and citric acid were used as the stimulus to explore the detailed quantitative equation of the strength variation between the sweet and sour taste sensations. The “close type” question was used to evaluate the sensation strength variation of sucrose sweet taste under citric acid background and citric acid sour taste under sucrose background. After that, according to Weber–Fechner law, the quantitative equation of the strength variation based on the absolute threshold and weber fraction were established. Additionally, equation calculation and human sensory evaluation comparison analysis were performed to verify the strength variation equation of synergism and antagonism effect based on sweet–sour taste.

## Results

### Sucrose sweet taste sensory strength variation model establishment

**Fig. 1:**
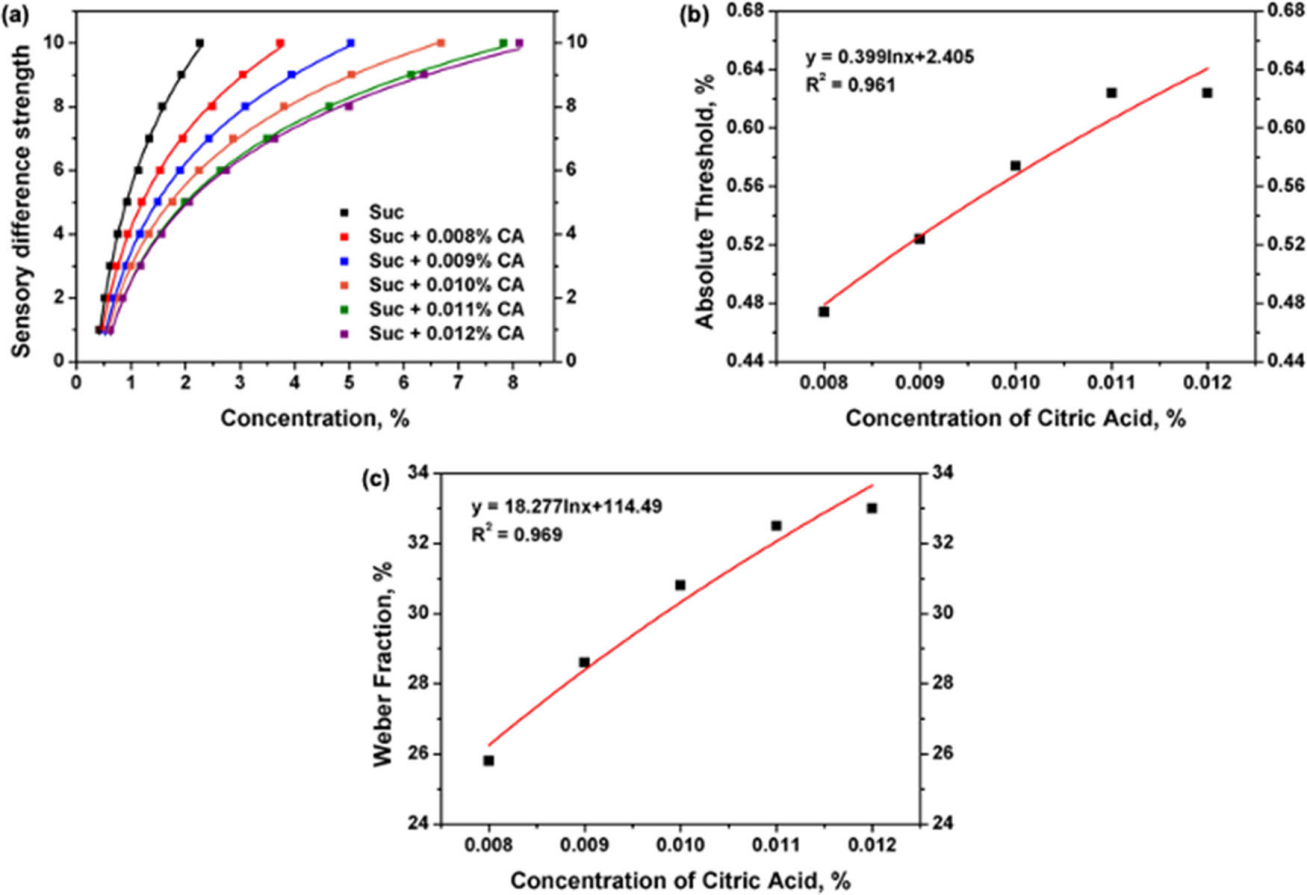
Sucrose taste sensation curves in different concentration of citric acid solutions. **a** Showed the sweet sensory difference strength of sucrose; **b** showed the relationship between sucrose absolute threshold value and citric acid concentration; **c** showed the relationship between sucrose Weber fraction and citric acid concentration.

According to the absolute threshold sensory analysis and difference threshold sensory analysis, the absolute threshold and difference threshold values for sucrose under different concentrations of citric acid solution background could be obtained. Taking the sucrose absolute threshold and difference threshold values as the *X*-axis and the sensory difference strength as the *Y*-axis, the sucrose sweet sensory difference strength curves could be plotted. The curves under 0.008%, 0.009%, 0.010%, 0.011%, and 0.012% of citric acid solutions are shown in Fig. 1a.

By fitting the data in Fig. 1a, it can be found that all the sucrose sweet sensory difference strength curves under different citric acid solutions are logarithmic curves, and all have a high correlation (*R*^2^ > 0.99). The above results indicate that, under the citric acid background, the sucrose sweet taste sensation is still in accordance with the Weber–Fechner law.

To research the variation effect of citric acid on sucrose sweet sensation more clearly, each weber fraction of the five sweet sensory difference strength curves was calculated via the difference threshold values. The absolute threshold and weber fraction results are listed in Table 1 and shown in Fig. 1b and c.

**Table 1:** The absolute threshold values and weber fraction of sucrose in different concentrations of citric acid solutions.

Name	Concentration of citric acid, %					
	0	0.008	0.009	0.010	0.011	0.012
Absolute threshold, %	0.424	0.474	0.524	0.574	0.624	0.624
Weber fraction*, %	20.5	25.8	28.6	30.8	32.5	33.0

*The weber fraction is the ratio of difference threshold and original stimulus concentration.

According to the Weber–Fechner law, based on the absolute threshold and weber fraction, the whole human sensory strength curve could be established. Therefore, by fitting the data of Fig. 1b, the sucrose absolute threshold value under different citric acid background could be calculated according to the following equation:

1 $$AT_{suc} = 0.399 \times \ln C_{ca} + 2.405$$

Where $AT_{suc}$ was the sucrose absolute threshold, the $C_{ca}$ was the citric acid concentration.

By fitting the data of Fig. 1c, the sucrose weber fraction value under different citric acid background could be calculated according to the following equation:

2 $$W_{suc} = 18.277 \times \ln C_{ca} + 114.49$$

Where $W_{suc}$ was the sucrose weber fraction, the $C_{ca}$ was the citric acid concentration.

Based on the Weber–Fechner law and our previous results^18,22^, the relationship between sucrose sweet sensory difference strength ($S_{suc}$) and its corresponding sucrose concentration ($C_{suc}$) would conform to the following derivation:

When the $S_{suc}$ was 1, $C_{suc1} = AT_{suc}$

When the $S_{suc}$ was 2, $C_{suc2} = C_{suc1} \times (1 + W_{suc})$

When the $S_{suc}$ was 3, $C_{suc3} = C_{suc2} \times \left( {1 + W_{suc}} \right) = C_{suc1} \times \left( {1 + W_{suc}} \right) \times \left( {1 + W_{suc}} \right) = C_{suc1} \times (1 + W_{suc})^2$

…

When the $S_{suc}$ was 3, $C_{sucs} = C_{suc1} \times (1 + W_{suc})^{s - 1}$

Therefore,

3 $$S_{suc} = \log _{\left( {1 + W_{suc}} \right)}\left( {\frac{{C_{sucs}}}{{C_{suc1}}}} \right) + 1$$

Substitute Eqs. (1) and (2) into Eq. (3), the sucrose sweet sensory difference strength under different citric acid background could be calculated according to the following equation:

4 $${{{\mathrm{S}}}}_{C_{suc}} = \log _{\left( {2.1449 + 0.1828\ln C_{ca}} \right)}\left( {\frac{{C_{suc}}}{{0.399\ln C_{ca} + 2.405}}} \right) + 1$$

Where $S_{C_{suc}}$ was the sucrose sweet sensory difference strength, the $C_{suc}$ was the sucrose concentration, the $C_{ca}$ was the citric acid concentration.

### Citric acid sour taste sensory strength variation model establishment

**Fig. 2:**
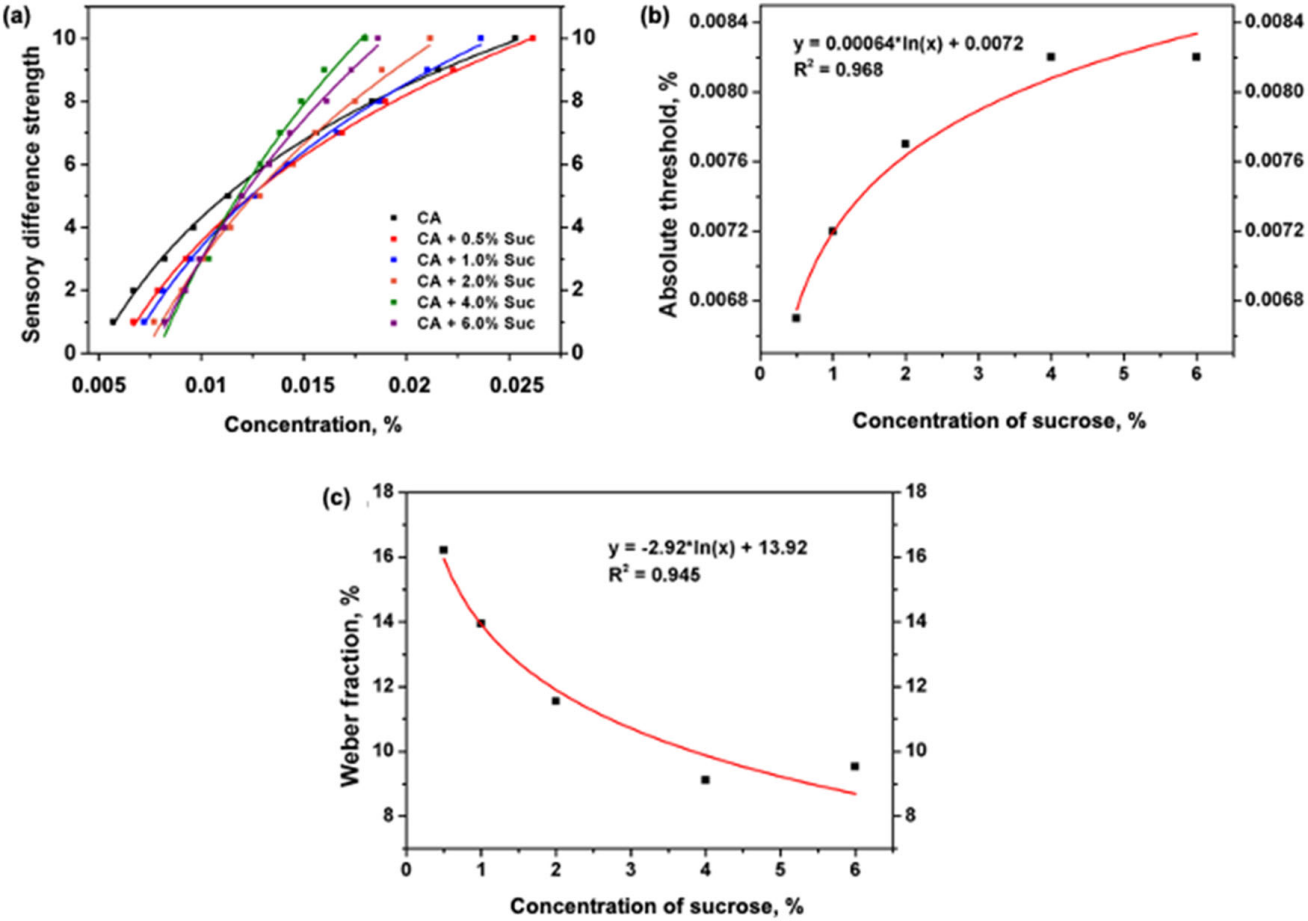
Citric acid taste sensation curves in different concentration of sucrose solutions. **a** Showed the sour sensory difference strength of citric acid; **b** showed the relationship between citric acid absolute threshold value and sucrose concentration; **c** showed the relationship between citric acid Weber fraction and sucrose concentration.

By using the same method, the citric acid sour sensory difference strength curves under 0.5%, 1.0%, 2.0%, 4.0%, and 6.0 % of sucrose solutions are shown in Fig. 2a. By fitting the data in Fig. 2a, it can be found that all the citric acid sour sensory difference strength curves under different sucrose solutions are logarithmic curves, and all have a high correlation (*R*^2^ > 0.99). These results indicate that, under the sucrose background, the citric acid sour sensation is still in accordance with the Weber–Fechner law.

Each weber fraction of the five sour sensory difference strength curves was calculated via the different threshold values. The absolute threshold and weber fraction results are listed in Table 2 and shown in Fig. 2b and c.

**Table 2:** The absolute threshold values and Weber fraction of citric acid in different concentrations of sucrose solutions.

Name	Concentration of sucrose, %					
	0	0.5	1.0	2.0	4.0	6.0
Absolute threshold, %	0.0057	0.0067	0.0072	0.0077	0.0082	0.0082
Weber fraction*, %	17.96	16.21	13.95	11.55	9.11	9.53

By fitting the data of Fig. 2b, the citric acid absolute threshold value under different sucrose background could be calculated according to the following equation:

5 $$AT_{ca} = 0.00064 \times \ln C_{suc} + 0.0072$$

Where $AT_{ca}$ was the citric acid absolute threshold, the $C_{suc}$ was the sucrose concentration.

By fitting the data of Fig. 2c, the citric acid weber fraction value under different sucrose background could be calculated according to the following equation:

6 $$W_{ca} = - 2.92 \times \ln C_{suc} + 13.92$$

Where $W_{ca}$ was the citric acid weber fraction, the $C_{suc}$ was the sucrose concentration.

Based on Eqs. (5) and (6), the citric acid sour sensory difference strength under different sucrose background could be calculated according to the following equation:

7 $${{{\mathrm{S}}}}_{C_{ca}} = \log _{\left( {1.1392 - 0.0292\ln C_{suc}} \right)}\left( {\frac{{C_{ca}}}{{0.00064\ln C_{suc} + 0.0072}}} \right) + 1$$

Where $S_{C_{ca}}$ was the citric acid sour sensory difference strength, the $C_{suc}$ was the sucrose concentration, the $C_{ca}$ was the citric acid concentration.

### Sweet and sour taste sensory strength variation model verification

**Fig. 3:**
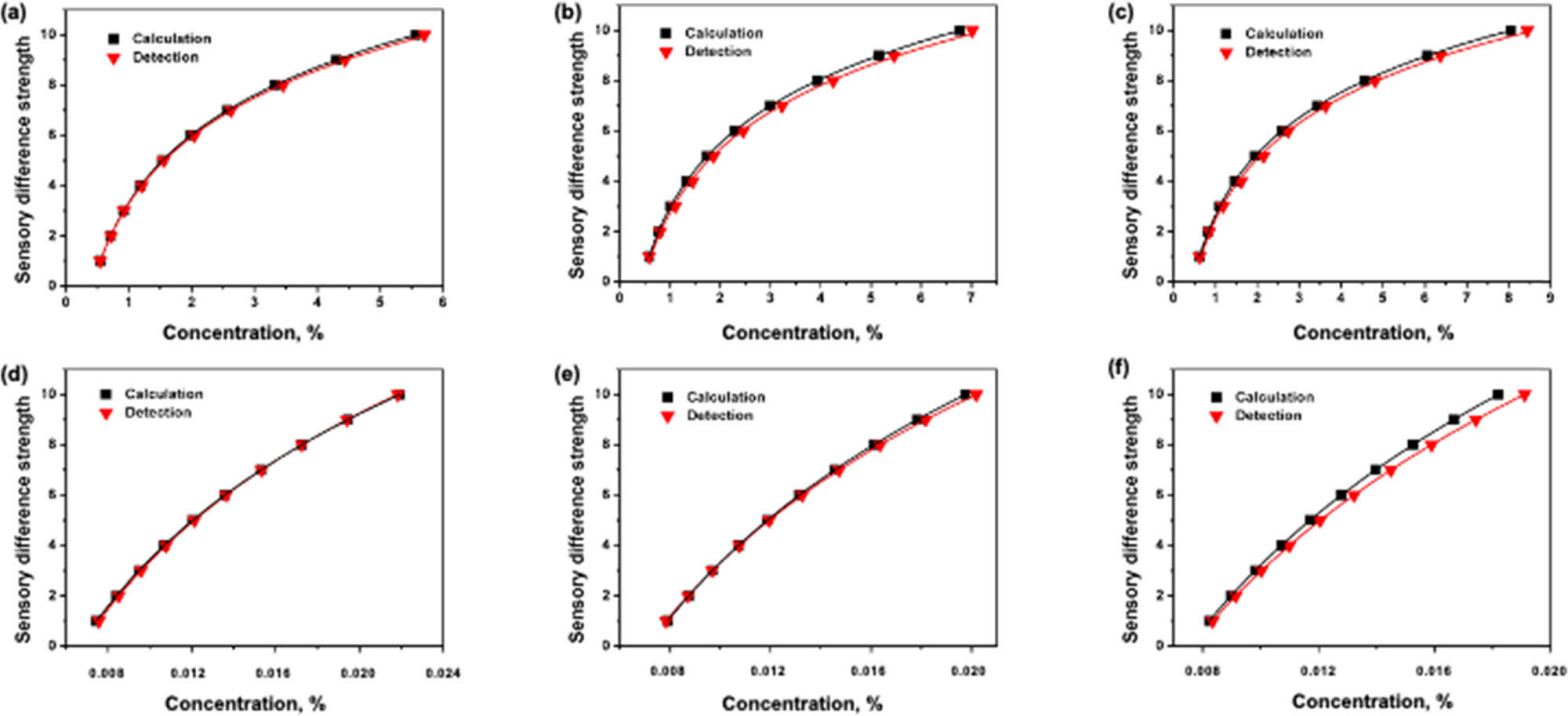
Sweet and sour taste sensory strength variation models verification comparison results. **a** Showed the sweet sensory difference strength of sucrose in 0.0095% of citric acid solution; **b** showed the sweet sensory difference strength of sucrose in 0.0105% of citric acid solution; **c** showed the sweet sensory difference strength of sucrose in 0.0115% of citric acid solution; **d** showed the sour sensory difference strength of citric acid in 1.5% of sucrose solution; **e** showed the sour sensory difference strength of citric acid in 3% of sucrose solution; **f** showed the sour sensory difference strength of citric acid in 5% of sucrose solution.

The sweet and sour sensory difference strength curves under different backgrounds were obtained from the human sensory evaluation and the model established in Eqs. (4) and (7). Then, the relative error analysis was performed on the two categories of curves to verify the accuracy of sweet and sour taste sensory strength variation. The results are shown in Fig. 3 and listed in Table 3.

**Table 3:** The detection and calculation comparison results of sucrose and citric acid SSTVM.

Sensory strength	Concentration of sucrose, %						Concentration of citric acid, %					
	0.0095% of citric acid		0.0105% of citric acid		0.0115% of citric acid		1.5% of sucrose		3.0% of sucrose		5.0% of sucrose	
	Sensory evaluation	Calculation	Sensory evaluation	Calculation	Sensory evaluation	Calculation	Sensory evaluation	Calculation	Sensory evaluation	Calculation	Sensory evaluation	Calculation
1	0.548	0.545	0.587	0.585	0.624	0.624	0.0076	0.0075	0.0078	0.0079	0.0083	0.0082
2	0.709	0.716	0.770	0.804	0.829	0.859	0.0085	0.0084	0.0087	0.0087	0.0092	0.0090
3	0.917	0.920	1.011	1.105	1.102	1.180	0.0096	0.0095	0.0097	0.0097	0.0100	0.0098
4	1.187	1.210	1.326	1.453	1.464	1.623	0.0108	0.0107	0.0108	0.0107	0.0110	0.0107
5	1.535	1.555	1.740	1.867	1.945	2.150	0.0121	0.0120	0.0119	0.0119	0.0121	0.0117
6	1.987	2.045	2.283	2.456	2.584	2.740	0.0136	0.0136	0.0133	0.0131	0.0132	0.0128
7	2.570	2.628	2.995	3.229	3.434	3.630	0.0153	0.0153	0.0147	0.0146	0.0145	0.0140
8	3.325	3.455	3.930	4.246	4.562	4.809	0.0173	0.0173	0.0164	0.0161	0.0159	0.0153
9	4.302	4.439	5.157	5.455	6.062	6.371	0.0194	0.0195	0.0182	0.0178	0.0174	0.0167
10	5.567	5.704	6.766	7.009	8.054	8.440	0.0218	0.0219	0.0202	0.0197	0.0191	0.0182
Average relative error, %	0.47						1.56					

In each figure of Fig. 3, the curves consist of the triangle and square symbol represent the human sensory evaluation and calculation results, respectively. For sucrose under citric acid background (as shown in Fig. 3(a) to (c)) and (as shown in Fig. 3(d) to (f)), the evaluation and calculation curves all exhibited the same logarithmic trend and had high degree of coincidence. Meanwhile, Table 3 lists all the acid difference threshold values of sucrose under citric acid background and citric acid under sucrose background by evaluation and calculation.

## Discussion

Compared to the pure sucrose, the sucrose sweet absolute threshold and difference threshold values were changed because of the citric acid existence. The sucrose absolute threshold increased firstly and then stabilized with the increase of citric acid concentration. The sucrose absolute threshold was 0.424% without citric acid and 0.624% when the citric acid concentration was 0.011% and 0.012%. This indicated that citric acid would decrease the ability of human to detect the initial sweetness of sucrose. Meanwhile, the sucrose weber fraction increased synchronously with the increase of citric acid concentration. The sucrose weber fraction was 20.5% without citric acid and 33.0% when the citric acid concentration was 0.012%. This suggested that citric acid would reduce the sensitivity of humans to detect the sucrose sweetness variation caused by its increased concentration.

On the other hand, compared to the pure citric acid, the absolute threshold and difference threshold values were changed because of the sucrose existence. The citric acid absolute threshold increased firstly and then stabilized with the increase of sucrose concentration. The citric acid absolute threshold was 0.0057% without sucrose and 0.0082% when the sucrose concentration exceeded 4.0%. This indicated that sucrose would decrease the ability of human to detect the initial sourness of citric acid. Meanwhile, the Weber fraction decreased firstly and then stabilized with the increase of sucrose concentration. The Weber fraction was 17.96% without sucrose and 9.1% when the citric acid concentration was more than 4.0%. This indicated that sucrose would increase the sensitivity of humans to detect the sourness variation caused by citric acid increased concentration.

Until now, the reports on the mechanism of sweet–sour interaction are rare. In the peripheral taste bud, sweet and sour taste are recognized by type II and type III taste cells, respectively. Taste cells communicate with each other within taste bud via paracrine neurotransmitters. Type II taste cells release ATP, adenosine, and acetylcholine, which exert excitatory transmitters to type III cells or themselves. Type III taste cells secret GABA and serotonin, which are inhibitory to type II cells. These interactions may explain the interactive response during sweet–sour stimulation.

Based on the above results, we named the Eqs. (4) and (7) as sweet–sour taste sensory strength variation model (SSTVM), which could be used to explain the human sensory sweetness and sourness interactive response, based on synergy and antagonism effect of sucrose and citric acid. As the SSTVM shows, the base and power values of sweetness and sourness interaction equations would be changed by the sweet and sour taste substances. Due to the variation of base and power values, the influenced sweetness and sourness after interaction could be calculated.

To verify the SSTVM, three concentration levels (0.0095%, 0.0105%, and 0.0115%) of citric acid solutions and three concentration levels (1.5%, 3.0%, and 5%) of sucrose solutions were used as the two models’ verification background, respectively. The average relative error values between evaluation and calculation were 0.47% and 1.56%. Meanwhile, the average relative root means squared error values were 0.16% and 4.1%. These results indicate that the absolute threshold values, difference threshold values and sweet–sour taste sensation strength of sucrose and citric acid calculated by the SSVTM established in this study have a high accuracy ratio. Therefore, by using SSVTM, human sensory sweetness and sourness interactive response based on the synergy and antagonism effect could be quantitively obtained.

According to SVVTM, the sweet–sour taste sensation interaction would be perceived more reliable. It would be helpful to provide a more suitable sweet/sour substance concentration in the food industry and human health management, including food additives usage, beverage and juice formula, and obesity therapy. In the intelligent sensory science research area, the SVVTM would be used to provide a more approximate human sensation reference to the taste sensors and electronic tongue.

## Methods

### Test samples preparation

Sucrose and citric acid were purchased from Sinopharm Chemical Reagent Co., Ltd (China). All the chemical reagents were used as received. All solutions were prepared using ultrapure water, the resistivity of 18.2 MΩ·cm by using the Millipore system.

The sucrose’s absolute threshold test samples and its compared samples were prepared into solutions in the different concentrations of citric acid solutions. The citric acid’s absolute threshold test samples and its compared samples were prepared into solutions in the different concentrations of sucrose solutions. The test samples concentration were wt. by volume and were listed in Supplementary Table 1. Before the sensory analysis, all the samples were kept at 20 °C.

The sucrose test samples of difference threshold and its compared samples were prepared into solutions in the different concentrations of citric acid solutions. In the sucrose’s first difference threshold test experiment, the compared sample concentration was its absolute threshold value. The sucrose’s first difference threshold test sample concentrations were 120%, 125%, 130% and 135% of its compared sample concentration. In the sucrose’s second difference threshold test experiment, the compared sample concentration was its first difference threshold value which was determined by the last experiment. The sucrose’s second difference threshold test sample concentrations were 120%, 125%, 130% and 135% of its compared sample concentration. The sucrose’s third to ninth difference threshold compared and test samples were prepared by the above analogy. All the sucrose test samples concentration were listed in Supplementary Table 2.

The citric acid’s difference threshold test samples and its compared samples were prepared into solutions by the different concentrations of sucrose solutions. The citric acid’s each difference threshold test samples concentrations were 105%, 110%, 115% and 120% of its compared sample concentration. Except for the above concentration setting, sample preparation method was the same as sucrose’s test samples. All the citric acid’s test samples concentration were also listed in Supplementary Table 2.

Before the sensory analysis, all the sucrose and citric acid test samples were kept at 20 °C.

### Sensory panel

The sensory panel consisted of 32 experienced assessors (sixteen males and sixteen females, from 20 to 35 years old), based on the Chinese national standard (GB/T 16291.1-2012: Sensory analysis—General guidance for the selection, training and monitoring of assessors—Part 1: Selected assessors). All subjects were told the purpose of this study and trained for two weeks (once per day) based on the following features:

(1) sensory evaluation terminology training, such as absolute threshold and difference threshold.

(2) basic taste sensation distinguishing, all the distinguishing tests should be correct.

(3) triangle test method and paired comparison test method training.

(4) the taste intensity evaluation relative error of the same concentration should be less than 10%.

All the assessors were asked not to smoke or eat at least a half-hour before each sensory evaluation experiment.

### Absolute threshold sensory analysis

The absolute threshold sensory analysis was based on the triangle test method which was mentioned in our previous work^18,22^. The absolute threshold values of sucrose under 0.008%, 0.009%, 0.010%, 0.011% and 0.012% of citric acid solution were obtained. The absolute threshold values of citric acid under 0.5%, 1.0%, 2.0%, 4.0% and 6.0% of sucrose solution were obtained through the same method.

### Difference threshold sensory analysis

The difference threshold sensory analysis was based on the paired comparison test method mentioned in our previous work^18^. The difference threshold values of sucrose under 0.008%, 0.009%, 0.010%, 0.011% and 0.012% of citric acid background were obtained. The difference threshold values of citric acid under 0.5%, 1.0%, 2.0%, 4.0% and 6.0% of sucrose background were obtained through the same method.

### Sweet and sour taste sensory strength variation models establishment

The sweet sensory difference strength curve was plotted with ab absolute threshold value and nine difference threshold values for sucrose under different concentrations of citric acid solution background as the *X*-axis and the sensory difference strength as the *Y*-axis. The variation equation of absolute threshold value, Weber fraction caused by different citric acid concentrations could be obtained via the sweet sensory difference strength curve. Additionally, the sweet taste sensory strength variation model of sucrose could be established via the variation equation of absolute threshold value and weber fraction, based on the Weber–Fechner law.

By using the same procedure, the sour taste sensory strength variation model of citric acid could be also established.

### Sweet and sour taste sensory strength variation models verification

The 0.0095%, 0.0105% and 0.0115% of citric acid solution were used as sweet taste sensory strength variation model verification background. The sweet sensory difference strength curves of sucrose under different backgrounds were obtained from the human sensory evaluation and the model established in section 2.6. then, the relative error analysis was performed on the two categories of curves to verify the accuracy of sweet taste sensory strength variation.

1.5%, 3.0% and 5% of sucrose solution were used as background, and the sour taste sensory strength variation model was verified by using the same procedure mentioned above.

## Supplementary information

Supplementary Table

## Data Availability

The authors declare that all data supporting the findings of this study are available in the paper and Supplementary Information.
