# Supplementary material for: Sensory sweetness and sourness interactive response of sucrose-citric acid mixture based on synergy and antagonism
Source: NPJ Sci Food. 2022 Jul 19;6:33. doi: 10.1038/s41538-022-00148-0 (PMC9296459; doi:10.1038/s41538-022-00148-0)
Supplement: Supplementary file 1 — Supplementary Table [file 41538_2022_148_MOESM1_ESM.pdf]

Supplementary Table 1 Absolute Threshold Compared and Test Samples of Sucrose and Citric acid

Sample	Compared sample	Sample concentration, %				
		Sample 1	Sample 2	Sample 3	Sample 4	Sample 5
Sucrose	0.008% citric acid solution	0.45	0.50	0.55	0.60	0.65
	0.009% citric acid solution	0.45	0.50	0.55	0.60	0.65
	0.010% citric acid solution	0.45	0.50	0.55	0.60	0.65
	0.011% citric acid solution	0.45	0.50	0.55	0.60	0.65
	0.012% citric acid solution	0.45	0.50	0.55	0.60	0.65
Citric acid	0.5% sucrose solution	0.0060	0.0065	0.0070	0.0075	0.0080
	1.0% sucrose solution	0.0065	0.0070	0.0075	0.0080	0.0085
	2.0% sucrose solution	0.0070	0.0075	0.0080	0.0085	0.0090
	4.0% sucrose solution	0.0075	0.0080	0.0085	0.0090	0.0095
	6.0% sucrose solution	0.0075	0.0080	0.0085	0.0090	0.0095

Supplementary Table 2 Difference Threshold Compared and Test Samples of Sucrose and Citric acid

Difference threshold	Sample concentration, %				
	Compared sample	Sample 1	Sample 2	Sample 3	Sample 4
1st	Absolute threshold value	120%[a], 105%[b] of the compared sample concentration	125%[a], 110%[b] of the compared sample concentration	130%[a], 115%[b] of the compared sample concentration	135%[a], 120%[b] of the compared sample concentration
2nd	1st difference threshold value				
3rd	2nd difference threshold value				
4th	3rd difference threshold value				
5th	4th difference threshold value				
6th	5th difference threshold value				
7th	6th difference threshold value				
8th	7th difference threshold value				
9th	8th difference threshold value				

[a] means the ratio of sucrose samples concentration.

[b] means the ratio of citric acid samples concentration.
